# Managing Organizational Inertia: Indonesian Family Business Perspective

**DOI:** 10.3389/fpsyg.2022.839266

**Published:** 2022-05-19

**Authors:** Teofilus Teofilus, Elia Ardyan, Timotius F. C. W. Sutrisno, Sabar Sabar, Verrell Sutanto

**Affiliations:** ^1^Faculty of Business and Management, School of Business and Management, Universitas Ciputra, Surabaya, Indonesia; ^2^Faculty of Business and Management, School of Business and Management, Universitas Ciputra, Makasar, Indonesia; ^3^Department of Visual Communication Design, Sepuluh Nopember Institute of Technology, Surabaya, Indonesia

**Keywords:** cynicism about organizational change, organizational inertia, empowering leadership, attribution theory, family business

## Abstract

The ability to transform on a regular basis is critical in the effort to adapt to external challenges; however, changes to an organization’s fundamental characteristics may increase the likelihood of failure. Because of this, organizational restructuring efforts appear to engender cynicism, which appears to be one of the most significant obstacles facing contemporary businesses, particularly in this area. Organizational inertia is the term used to describe this aversion to change, as well as the desire to maintain the current status quo. A new organizational culture capable of combating the incidence of organizational stagnation is required by massive social, economic, and technological difficulties, and firms that employ the concept of empowering leadership will be able to meet these challenges. For the purposes of this study, a framework for discussing the phenomena of organizational cynicism was developed and implemented.

## Introduction

In a constantly changing business environment, long-term success necessitates not just the ownership of difficult-to-replicate assets, but also the possession of one-of-a-kind and outstanding dynamic talents. Several scholars claim that this competitive advantage will be realized in the area of human capital management if the organization is able to design the connectivity of human resources inside it under the auspices of a high-performing work system (Quratulain and Al-Hawari, 2021; Nguyen et al., 2022). Organizations strive to adjust their strategy in order to face the challenges posed by changes in the company life cycle (Santiago, 2015). Organizations that are able to adapt to new markets, processes, and technology are known as entrepreneurial enterprises (Sharma et al., 2012; Santiago, 2015). For firms confronting changes in the organizational life cycle, innovation appears to be a sensible course of action. Organizations, on the other hand, do not always innovate, and some can fall into a condition of immobility. In family enterprises, inertia is also common (Chirico and Nordqvist, 2010; Chirico and Salvato, 2016). Inertia increases in families that stay closed and paternalistic. The business family becomes rigid and resistant to change as a result of the paternalistic mindset, whereas the entrepreneurial drive encourages innovation (Chirico and Nordqvist, 2010). The refusal to modify the core of the organization is referred to by Mallette and Hopkins (2013) as structural inertia, biased management cognition (Gilbert, 2005). Cynicism about change and even hostility to change stems from the organization’s closed, paternalistic culture and refusal to change its essential values. In addition, some research have found a link between cynicism and organizational inertia. Huang et al. (2013) claimed that inertial organizational conditions will impede the implementation of organizational strategy, making organizational sustainability uncertain (DeCelles et al., 2013; Fernhaber and Li, 2013).

According to research on organizational inertia, there is a significant internal propensity towards similarity, which can inhibit employees’ ability to produce novel ideas (AlKayid et al., 2022). Visionary leadership (AlKayid et al., 2022), flexible budgeting (Oyadomari et al., 2018), skewed management cognition, a lack of incentive to change, or challenges in redeploying business resources (Gilbert, 2005; Hoppmann et al., 2019) are some of the antecedent variables. The character of cynicism as a serious barrier to change (Reichers et al., 1997), cynicism as something that develops, is destructive, and is possible to sabotage (DeCelles et al., 2013), and the reluctance of cynical employees to participate in change are all factors considered in this study (Islam et al., 2020). Cynical personnel have a passive attitude toward change, which leads to organizational stagnation in the form of incapacity to implement internal adjustments in the face of large external changes (Gilbert, 2005).

In recent years, the topic of organizational cynicism has become an intriguing subject for further exploration. Given the strong correlation between cynicism and an employee’s professionalism (Bang and Reio, 2017), cynicism, particularly in Indonesia, is worth investigating further. Indonesia is a collectivist country with a high power distance (Aslam et al., 2016), and its response to cynicism is quite unique, promoting tolerance and respect for others and concealing cynicism within an organization. Milliken et al. (2003) characterized cynicism as “silent cynicism” in a study they conducted.

When an organization changes, the comfort and stability of the workplace are frequently disrupted. This is because when an organization changes, it creates uncertainty and discomfort in the work environment, which contributes to employee cynicism (Oreg, 2006; Aslam et al., 2016). Numerous studies on employee behavior in response to organizational change have been conducted over the last few decades (Reichers et al., 1997; Wanous et al., 2000; Brown and Cregan, 2008; Grama and Todericiu, 2016). The study agrees that employee cynicism will have an effect on the organization’s performance (Bouckenooghe et al., 2014, 2021; Cinite and Duxbury, 2018).

According to several studies, it is believed that by involving employees, an organization’s cynicism can be reduced. Numerous studies indicate that an organization’s cynicism can be reduced by involving employees in the planning process, conducting performance evaluations, and being willing to admit mistakes (Ahearne et al., 2005; Stanley et al., 2005). Employees who feel empowered are more likely to take proactive measures and support the change process; additionally, superiors must demonstrate their recognition of employees’ competence, as this fosters employees’ confidence and security in the organization by allowing them to work independently and providing support. Increasing the capacity of an organization in order to ensure its long-term viability (Jung et al., 2020).

There is a wealth of research demonstrating the link between HPWS and organizational performance. This study will look at HPWS from a variety of angles. The study’s link between strategic human resource management and psychology is in the establishment of empowering leadership mechanisms to manage cynicism and organizational inertia. Confidence in the organization and senior management is increased by preparing followers to accept potential negative experiences during any transformation effort (Zhang and Bartol, 2010; Van Bockhaven et al., 2015; Hao et al., 2017; Lee et al., 2018). As a result, empowered leadership can assist followers in discovering purpose in their job and establishing a sense of security within the organization, while simultaneously fostering creativity and lowering hazardous defensive behavior. As a result, empowering leaders has a beneficial effect on their followers’ risk-taking behavior (Jung et al., 2020). Empowering leaders through development support for the technical and managerial skills required of followers enables followers to initiate change-related activities (Lorinkova and Perry, 2017; Kundu et al., 2019).

This research employs a social cognitive theory approach, which Bandura (1997) asserts consistently demonstrates successful self-leadership abilities and other desirable work-related behaviors. The learning model is a component of social cognition theory and the idea of triadic reciprocity, which asserts that an individual’s cognitive processes, behavior, and environmental impacts are all interconnected (Amundsen and Martinsen, 2014; Newman et al., 2018). Empowerment improves the work environment and increases individual motivation to work indirectly by providing autonomy and development support to lower levels in an organization where decisions can be made, particularly in terms of implementing creative and innovative changes that address business needs. Will eventually result in the creation of a sustainable business (Turi et al., 2019; Lin and Ling, 2021). The interaction of the three triadic reciprocal components is predicted to expand the usage of social cognition theory in the setting of family business. Three components: personal (cynicism about change as a negative attitude among organizational members), environmental (empowering leadership as a positive environment the leader attempts to create), and behavioral (cynicism about change as a negative attitude among organizational members; organizational inertia as a form of behavior after going through the learning process). In this context, empowering leadership is a variable that reduces cynicism and reduces the impact of cynicism on organizational inertia changes in family businesses (Li and Yuan, 2017; Meng-Hsien et al., 2018).

## Theoretical Background

### Social Cognitive Theory

“Social Cognitive Theory” is a foundational theoretical framework that has been shown to be effective in comprehending and explaining behavior (Bandura, 1997). The individual (person), the environment (environment), and individual behavior (behavior) all have a reciprocal link, which is referred to as (triadic) reciprocal determinism (or triadic reciprocal model of causality; Bandura, 1997; Eslami and Melander, 2019). The essence of this theory is that humans acquire the ability to model through observation and imitation, which they subsequently use when behaving or acting. Humans react by utilizing their capacity for thought, symbolism, and anticipation (outcome reaction). It is critical to emphasize the relationship between individual characteristics, group values, attitudes, and behavior throughout organizational change (Bandura, 1997). This theory is predicated on the following assumptions: humans view humans intrinsically, not as good or bad, but as a result of experience with the potential for all kinds of behavior; humans are capable of conceptualizing and controlling their behavior; humans are capable of acquiring new behavior; and humans can influence the behavior of others just as their behavior is influenced by others (Ilgen et al., 2005; van Zundert et al., 2010) proposed four critical parts to this theory in order to explain it: observational learning (modeling), self-regulation, self-efficacy, and reciprocal determinism. Cynicism toward university changes develops when new obligations are not accompanied by equitable justice. The justice approach is supposed to be capable of fostering an environment conducive to learning, self-evaluation, and constructive behavior (Yim et al., 2017).

### Cynicism About Organizational Change

Cynicism is essentially the end result of a preceding process (Wanous et al., 2004; Grama and Todericiu, 2016; Schraeder et al., 2016; Bakari et al., 2019). According to Dean et al. (1998), there are five fundamental conceptualizations of cynicism: personality cynicism, societal or institutional cynicism, occupational cynicism, employee cynicism, and skepticism about organizational transformation. Cynicism is defined as a person’s lack of trust in others or their perception of others as dishonest, unsocial, immoral, ugly, or even vicious (Abugre, 2017; Rayan et al., 2018; Schmitz et al., 2018; Zeidan and Prentice, 2022). To be more precise, this research will refer to “cynicism about organizational change” as a moderate attitude toward future organizational changes that includes pessimism about their success, based on the perception that changes are prone to failure and the belief that change agents are incompetent (Wanous et al., 2004).

Wanous et al. (2004) coined the term “cynicism about organizational change,” which refers to a genuine loss of trust in change agents as a result of a history of change initiatives that were not fully or obviously effective. Additionally, because those who are cynical about organizational change may rationalize away knowledge gaps with the rationale that things must not have gone well, ineffectiveness and failure foster pessimistic attitudes, which further inhibit motivation to try again and become a significant impediment to change (James, 2005; Stanley et al., 2005; Abugre, 2017). It occurs despite the best intentions of those responsible for the change; even for rational decision makers who care about both employee well-being and their own reputations (Stanley et al., 2005; Walter and Cole, 2011; Neves, 2012). Cynicism about organizational change has previously been defined as a composite of three components: (a) pessimism about the success of future organizational change, (b) a dispositional attribution that those responsible for change are less motivated, incompetent, or both, and (c) a situational attribution (Wanous et al., 2000, 2004; Stanley et al., 2005). Pessimism is defined as an individual’s assessment of the likelihood that future organizational reforms will be effective. Meanwhile, dispositional attribution is concerned with the motivation and ability of organizational leaders, whereas situational attribution is concerned with circumstances beyond their control (Wanous et al., 2004). In the context of a family business, the term “successor” is not widely used. Leadership transformation is not a position that can be filled by random individuals, but rather by owner placement and direct appointment. Cynicism is critical to manage in this case because it is prone to occur in family businesses.

Additionally, the level of enthusiasm for new projects varies by individual and hierarchy. Changes may be viewed as fascinating challenges or as appropriate and timely responses to a changing environment; however, lower-level employees may regard them as incomprehensible and inexplicable actions because top-level management (parents in the business family) is typically conservative and lacks the capability to adapt to a changing environment (Brown and Cregan, 2008; Qian and Daniels, 2008; Scott and Zweig, 2016). Hourly workers expressed more cynicism about organizational change than executives did. Perhaps executives and managers believe they have a better understanding of upcoming plans and decision-making processes (Reichers et al., 1997). According to a previous empirical study conducted by (Stanley et al., 2005; Qian and Daniels, 2008; Grama and Todericiu, 2016; Bakari et al., 2019; Scott and Zweig, 2020), cynicism about organizational change is likely caused by a lack of general knowledge about what was happening in the workplace, a lack of communication and respect from the supervisor or union representative, a negative disposition, and a lack of opportunity for meaningful participation in decision-making.

According to Wanous et al. (2000), Cynicism about organizational change has two possible antecedents: negative affectivity as a personality trait and organizational factors. For example, prior exposure to change may predispose some employees to cynicism, which includes pessimism about the success of change initiatives. The supervisor’s role efficacy includes conveying information, listening effectively, being available, and showing concern. Participation in decision-making is the third organizational factor that has been linked to cynicism about organizational change. Employee cynicism can be influenced by top management. Unless they are used as selection criteria, top management cannot influence personality traits (Wanous et al., 2000).

### Organizational Inertia

As previously stated by Hannan and Freeman (1984), Rumelt (1995), and Gilbert (2005), when an organization has structural inertia or a strong strategy, the organization is prone to resist adaptive adjustments to changes in the external environment and is more comfortable with the status quo. This is because an organization’s adaptation to a change will have an effect on the organization’s existing characteristics, such as its routine operating procedures, organizational structure, resource allocation methods, and decision-making procedures (Yi et al., 2016; Hoppmann et al., 2018; Zhen et al., 2021). Inertia in an organization results in a condensing of the organization’s operating mode and direction, reducing its flexibility (Hannan and Freeman, 1984; Godkin and Allcorn, 2008; Allcorn and Godkin, 2011; Sillic, 2019). Organizational inertia has two components: resource rigidity and routine rigidity (Gilbert, 2005). It is the inability of a company to change its resource investment pattern, while routine inflexibility is the lack of change in organizational processes and procedures for using invested resources. (Gilbert, 2005; Moradi et al., 2021).

In organizational literature, the terms organizational inertia and organizational flexibility are mutually exclusive. Flexibility has a number of advantages, and organizations that are more adaptable are more efficient. Inertia manifests itself in a variety of ways in organizations, including the suppression of valuable information within the organization, rigid rules, and an excessive commitment to the organization (Boyer and Robert, 2006; Dew et al., 2006). The organization is an open system that interacts with its surroundings and is self-sufficient. Closed communication and information channels cause an organization to be unaware of changes occurring around it, leading to its demise. Inflexible organizations and individuals are unable to adapt to changing environments. Individual stagnation leads to organizational inertia (Boyer and Robert, 2006; Hirschmann, 2021; Moradi et al., 2021).

### Empowering Leadership

By combining social cognitive theory and organizational inertia, this study sought to understand the relationship between cynicism about change and leader empowerment. Humans, according to SCT, are both environmental consumers and producers (Bandura, 2001). Humans’ ability to choose and control their own behavior through deliberate action is called organization (Bandura, 1989, 2001). SCT proposes five mechanisms for learning and shaping behavior. Observation, reflection, self-regulation, and symbolization are the mechanisms. To test the Empowering Leadership development intervention’s effectiveness in reducing cynicism, and thus unsafe behavior, we used SCT and the underlying mechanisms.

Employees’ work is valued, decision-making authority is increased, and unwanted factors such as harassment are eliminated (Zhang and Bartol, 2010). Enabling does not sum up “sharing power.” Empowered employees can self-manage to improve work psychological cognition. Furthermore, subordinate motivation should be considered holistically (Dong et al., 2015; Li et al., 2016; Kim et al., 2018b). Basically, empowerment is a matter of degree rather than absolute state, so the issue is managers’ ability to classify both decisions about who to empower and how much (Cheong et al., 2016; Lorinkova and Perry, 2017; Kim et al., 2018b). However, empowerment can also be seen as a mutually beneficial relationship between a leader and his subordinates (Qian et al., 2018; Muafi et al., 2019; Lin et al., 2022). Thus, it is critical to always improve team performance by encouraging problem-solving initiative, quick communication, and improved work-life balance. So Amundsen and Martinsen (2014) focus on power sharing, motivation support, and development support.

Power sharing is a basic application of employee empowerment. Its indirect link between self-leadership and freedom within bounds (e.g., encourage independent actions). According to (Cheong et al., 2016; Li et al., 2016; Qian et al., 2018), decision-making procedures distinguish consultation and delegation. Leaders engage subordinates in consultation before delegating authority and decision-making responsibility (Cheong et al., 2016, 2019). Kim et al. (2018a) noted that delegation provides real autonomy in decision making. To feel empowered, everyone must agree on their overall goal and what actions they can take to achieve it. Leaders must motivate subordinates to take initiative, make decisions, and lead themselves (Amundsen and Martinsen, 2014). Encourage subordinates to work toward self-determination and inspire them with goals (Jung et al., 2020; Lin et al., 2022). Employees believe that for them to feel positive and confident in their abilities, it is critical for’superleaders’ to approach subordinates with an open ear and listen to their ideas. As a result, it may foster autonomy and have a significant effect on motivation and efficacy. Additionally, we advise leaders to create a welcoming environment in which subordinates can discover their capabilities, inspire employees, and apply their abilities (Li et al., 2016; Jung et al., 2020). Empowering and inspiring leaders can inspire and create positive emotional states by demonstrating enthusiasm and belief in their future goals and prospects.

The last essential construct for empowering leadership according to Amundsen and Martinsen (2014) is development support, which explains the main characteristic of leaders is to serve as observable models for their subordinates (Cheong et al., 2016; Li et al., 2016). Model learning is a concept in social cognitive theory that implies a behavior can be learned or modified by observing others (learning by example). This is more likely because the models have status, power, success, and/or competence (Hao et al., 2017; Jung et al., 2020). So this study uses two empowering leadership dimensions. It describes how a leader empowers members to take initiative through delegation, coordination, and information sharing. This dimension describes how a leader can model and guide members to keep learning. To motivate and develop subordinates to work autonomously within the organization’s goals and strategies is a genuine concern of leaders (Amundsen and Martinsen, 2014).

### Sustainable Family Business

Family businesses must be built on a solid foundation of family meetings, respect, and communication. The first step toward family business sustainability is to understand the basics. Competitive advantages are typically fleeting in high-tech environments, whereas advantages may be more sustainable in low-tech environments (Weemaes et al., 2020). Thus, a family firm is defined as one that is “governed and/or managed with the intention of shaping and pursuing the business vision held by a dominant coalition of members of the same family or a small number of families in a manner that is potentially sustainable across generations of the family or families.” Family-owned enterprise Sustainability is defined as the capacity to recover, rebound, or revert to pre-existing conditions following the occurrence of problems or stresses (Gupta and Bhattacharya, 2016). Lee et al. (2013) were able to quantify an organization’s potential for sustainability (resilience) by examining adaptability measures such as managers’ perceptions of environmental risk, their willingness to seek information about environmental risks, the organization’s structure, their level of involvement in community planning activities, their level of compliance with continuity of operations planning, and whether the department has professional accreditation (Gupta and Bhattacharya, 2016).

According to the Sustainable Family Business (SFB) model, a sustainable family business is an integrated function of the family’s functionality and the business entity’s success (Kuruppuge and Gregar, 2018; Pitchayadol et al., 2018) and that each of these two components has a two-way influence on the other (Heck and Trent, 1999). Aldrich et al. (2021) established that social networks, including families, foster the establishment, growth, and transition of family businesses. Additionally, (Herrero and Hughes, 2019; Vecchio et al., 2019) discovered that the manner in which family members interact has a significant but inconsistent relationship with the family business’s continuity. This mode of interaction encompasses the negotiation process, everyone’s accessibility, each team member’s individuality, and routines.

## Hypotheses

Cynicism for change refers to the degree to which people are pessimistic about the future of change initiatives, as well as about their own management skills and abilities to bring about change success (Choi, 2011). Wanous et al. (2000) distinguish between two types of cynicism toward change: pessimism about the change itself and dispositional attributes that are associated with those who are responsible for implementing the change. Pessimism, on the other hand, is of particular interest because it is closely associated with generalizable individual attitudes. Comparatively, because they can relate to stakeholders other than management, such as trade union representatives, dispositional attributes lack the ‘focus specificity’ necessary to be practically useful in change management studies, and therefore are not practical in change management studies (Albrecht, 2002). Consequently, the current research will concentrate primarily on the cynical side’s cynicism regarding change.

Interestingly, cynicism about change appears to be a significant in the ability to successfully implement change, making this concept very intriguing. Change is invoked in individuals more frequently (and unsuccessfully) the more likely it is that they will express cynicism about the change (Brown et al., 2017). Employee engagement, on the other hand, in accordance with the aforementioned constructs, plays an important role in preventing cynicism from changing. It is possible to reduce the likelihood of change cynicism by sharing and communicating information while also involving individuals in the decision-making process. Nonetheless, when individuals are cynical about change, resistance to change is more likely to occur, increasing inertia at the individual level as a result (Stanley et al., 2005). Inertia can result from ignoring this individual’s opposition to the desired change, because individual support is required for the significant implementation of the intended change (Fernandez and Rainey, 2017). In order to successfully avoid inertia, it may be necessary to overcome this individual changing attitude.

Based on the findings and discussion above, the following hypotheses can be proposed:

*H1*: Cynicism about organizational change has a positive and significant effect on organizational inertia.

Even for highly successful businesses, inertia can lead to difficulties in adapting to new business methods. Moradi et al. (2021) demonstrate that the business management model is accompanied by risk and uncertainty, and that the inertia that exists in organizations that have had successful business models in the past leads to business model problems when accepting new business models. Reconfiguring a business model interacts with issues that must be addressed, such as: (1) overcoming inertia, (2) identifying multiple changes, and (3) adopting a new structure and selecting an appropriate approach to improvement. Because of organizational inertia and the resulting uncertainty, firms are unlikely to define their business model unless they are faced with a significant change in their industry or market. Even in cases where adaptation is obvious, the firm’s strategic direction and path dependencies are likely to make the process of adapting existing business models to new market demands or competitive threats more difficult and time-consuming (Vorbach et al., 2017). Therefore, it can be hypothesized that:

*H2*: Organizational inertia has a negative and significant effect on sustainable family business.

Cynicism about organizational change has a destructive effect on the organization, and it can even lead to acts of sabotage (DeCelles et al., 2013). Organizational inertia will be created as a result of cynicism about change as a result of a negative attitude (Huang et al., 2013). The development of organizational inertia (resources rigidity, processes rigidity, and path dependency) in a family business will result in the company’s inability to actualize the agility that is required in a rapidly changing business environment if allowed to continue (Huang et al., 2013). According to social cognitive theory, cynicism about organizational change is a personal trait that must be developed (Bandura, 1989). Furthermore, empowering leadership can be defined as a leader’s action in creating a favorable environment for initiated changes to take place (Lorinkova and Perry, 2017). Then, as a form of suppression, empowering leadership will be able to suppress cynicism about change as a result of its empowerment (Frazier et al., 2004; Li and Yuan, 2017). It is expected that the interaction of the two variables will act as a buffer, reducing the negative impact of cynicism on changes in organizational inertia (Huang et al., 2013). Therefore, this study formulated the following hypothesis:

*H3*: Empowering Leadership will be able to reduce the negative effect of CAOC on organizational inertia in family business in Indonesia.

## Materials and Methods

### Research Setting and Sampling Procedure

To address the study’s research question, the first step was to select a research sample representative of an organizational inertia phenomenon, specifically family businesses. Questionnaires were distributed to family business founders and members regarding research variables and changes in family business succession. After effectively tabulating the data, data aggregation, processing, and hypothesis testing are performed; additional discussion, as well as theoretical and practical implications, are produced after the findings are acquired. This is a quantitative study conducted using a cross sectional design, in which all measurements on each person are taken at the same time. The population of this study is Indonesian family-owned businesses. The sampling technique used in this study is non-probability sampling, which means that not all samples have an equal chance of being chosen as a sample. Meanwhile, this study’s sample selection technique is purposive sampling. To qualify as a family business, the owner/manager must have been in the business for at least 1 year or be actively engaged in the business for at least 6 h per week or at least 312 h per year while living with other family members. As a result, this study’s sample is limited to businesses that meet those criteria. The research sample is distributed throughout Indonesia and includes 31 family businesses operating in a variety of sectors or fields, including food and beverage, medicine, electronics, garment manufacturing, and the automotive industry. In total, 124 people were sampled for this study, including 31 leaders from various family businesses located throughout Indonesia and 93 members, three from each organization.

### Measurement

This study’s sample units are divided into two categories: leaders (top to middle management) and members (lower management). This study examines four variables, two of which are distributed to the family business leader and the rest to family business members. This study examines members’ cynicism about organizational change and empowering leadership, while measuring organizational inertia and family business sustainability. This study used a questionnaire to collect primary data, i.e., a prepared list of questions. The cynicism about organizational change variable has 16 operational items adapted from Wanous et al. (2004). The reasons for using dispositional cynicism in this study are (a) distrust of integrity, competence, and leadership motivation (common in family businesses); and (b) the data quality test results for pessimism and situational cynicism show that they do not pass the reliability test. For example, resource dependency, position reinvestment incentives, threat perception, contraction of authority, reduced experimentation, focus on existing resources and learning effects are all operational items of organizational inertia adapted from Gilbert (2005). This variable includes autonomous support (power sharing and motivation support) and development support, which are both adapted from Amundsen and Martinsen (2014).

Each leader is represented by 3 (three) members in each family business, implying that each business family must have a minimum of four members. The sample for this study included 31 family business leaders and 93 family business members or employees. Additionally, data aggregation was used to combine data collected from two distinct subjects in the family business. Aggregation of data is a two-step process. To begin, one or more data groups are identified based on the values in selected features (data grouping); second, the values in one or more selected values are aggregated for each group.

### Data Collection

The questionnaire generation process was carried out in two stages, referred to as double-back translation, in which operational items adapted from previous research were translated to Bahasa Indonesia and then back to English to avoid misinterpretation during the translation process. Additionally, the questionnaire was rechecked for informal fallacies such as double-barreled questions, which are questions that address multiple issues but allow for only one response. Meanwhile, the questionnaire used the Likert scale as a measurement tool in this study. The Likert scale is a useful indicator of a study with five (five) scales, as it simplifies the process of calculating results and makes responding easier for respondents (Sekaran and Bougie, 2016). After completion, the questionnaire was distributed to family businesses throughout Indonesia, with a leader and members representing each sample unit.

### Method of Analysis

This study’s data are processed using Multiple Moderated Regression (MMR). MMR is a statistical method for assessing the impact of moderation in a research model. The general procedure of this method is to examine the effect of the independent variable (X) on the dependent variable (Z) and the effect of the product (XZ) on the independent variable (Z). The independent variable’s effect on the dependent variable varies at intervals determined by the moderator variable (Hayes, 2018). This study’s goal was to examine how empowered leadership affected cynicism about organizational change, organizational inertia, and family business sustainability. The measurement model for this research was validated and reliability tested in advance. Validity was assessed using EFA, CFA, and PCA (PCA). The reliability test used Cronbach Alpha, Corrected Item Total Correlation, and Split-half testing. In addition, the *F*-test, coefficient of determination, and *t*-test results were examined in this study. The *F*-test was used to assess the significance of the regression model and the effect of all independent variables on the dependent variable. It was determined by the coefficient of determination (or *t*-test) whether or not each independent variable had a significant effect on the dependent variable.

## Results

### Measurement Validation

Validity is determined by the value of the outer loading, which according to Hair et al. (2017) has a cutoff of 0.500, whereas reliability is determined by the reference value of composite reliability and the AVE value, with a recommended CR value in the range of 0.700 and an AVE value greater than 0.500 (Fornell and Larcker, 1981). As shown in Tables 1 and 2, the overall value of the outer loading does not fall below the standard of 0.500, and the AVE value is also greater than 0.5. Thus, the data used in this study satisfy the validity assumption. Additionally, the composite reliability value is greater than 0.700, indicating that the data used is reliable. Additionally, as shown in Table 2, pro-change behavior is negatively correlated with pro-change cynicism.

**Table 1:** Validity and reliability result.

Code	Item	Outer loading	Reliability	AVE
SCA1	Child has a commitment to continue the family business	0.702	0.970	0.635
SCA2	Business does not just stop at the first generation	0.637		
SCA3	Family business always earns profit in the long term	0.674		
SCA4	Prospective successors are able to foster a sense of harmonization between siblings in running a family business	0.894		
SCA5	Prospective successors are able to foster a sense of harmonization with employees	1.011		
CAOC17	The people who are responsible for making improvements around here do not know enough about what they are doing	0.901	0.977	0.678
CAOC16	I’ve suspected that the leaders’ public statements reflect more spin than reality	0.917		
CAOC14	I marvel at the disparity between reality and the leaders’ claims	0.607		
CAOC9	I suspect the leaders is deliberately evasive in the things they say	0.865		
CAOC8	I have misgivings whether the leader is forthright regarding their actions	0.535		
CAOC18	Most of the people who are responsible for solving problems around here do not try hard enough to solve them	0.930		
CAOC19	The people responsible for making things better around here do not care enough about their jobs	0.939		
CAOC20	The people who are responsible for solving problems around here do not have the skills needed to do their jobs	0.789		
Empower20	My leader’s planning of his/her work is visible to me	0.723	0.994	0.586
Empower19	My leader lets me see how he/she organizes his/her work	0.850		
Empower16	My leader is enthusiastic about what we can achieve	0.384		
Empower15	My leader invites me to use my strong sides when needed	0.422		
Empower14	My leader recognizes my strong and weak sides	0.717		
Empower13	My leader listens to me	0.840		
Empower12	My leader is concerned that I work in a goal-directed manner	0.823		
Empower11	My leader makes me work towards goal attainment	0.766		
Empower10	My leader is concerned that I reach my goals	0.643		
Empower9	My leader encourages me to take initiative	0.751		
Empower8	My leader expresses positive attitudes related to me starting with my own defined tasks	0.674		
Empower7	My leader encourages me to start tasks on my own initiative	0.690		
Empower6	My leader discusses shared affairs with me	0.799		
Empower5	My leader talks with me about his/her own and my goals	0.763		
Empower4	My leader coordinates his/her goals with my goals	0.737		
Empower3	My leader gives me authority over issues within my department	0.827		
Empower2	My leader gives me power	0.708		
Empower1	My leader conveys that I shall take responsibility	0.713		
Empower21	I gain insights into how my leader arranges his/her work days	0.707		
Empower22	My leader shows me how I can improve my way of working	0.620		
Empower23	My leader guides me on how I can best do my work	0.894		
Empower24	My leader tells me about his/her own way of organizing his/her work	0.876		
Inertia1	The company did not succeed in implementing new products/services development	0.701	0.890	0.521
Inertia 3	New additional employees were not hired to work in the area of developing new products/services	0.590		
Inertia 8	Budget control was made tighter	0.606		
Inertia 15	The company is not seeking for higher efficiency in order to find synergies among several activities	0.861		
Inertia 16	The company did not learn and obtain new skills and experiences while implementing new products/services	0.755		
Inertia 17	The new product/service did not require new knowledge and skills from the company	0.752		
Inertia 18	It is difficult to “forget” the former success stories	0.751		

**Table 2:** Descriptive statistic.

Variable	SD	Mean	1	2	3	4
SCA	0.481	4.503	**0.678**			
CAOC	0.527	2.416	−0.107	**0.521**		
Empower	0.485	3.884	0.364^*^	−0.587^**^	**0.586**	
Inertia	0.524	2.248	−0.618^**^	0.276	−0.578^**^	**0.635**

Values on the diagonal are AVE. Values below the diagonal are inter-factor correlations.

* Correlation values are significant at *p* < 0.05.

** Correlation values are significant at *p* < 0.01.

### Data Analysis

We assessed the study’s construction and analysis level using a group-level analysis approach, with the family business as the unit of analysis. As a result, data collection from each unit to represent their respective groups is necessary. The RWG approach is used to merge individual group data into team-level group data (James et al., 1984; Walumbwa et al., 2017), with a minimum value of 0.700.

### Hypothesis Testing

The PROCESS macro is used to run SPSS to test the moderated mediation hypothesis (Hayes, 2018). In a more detailed model, we examined the impact of cynicism on changes in Cynicism (Cyndisp) on changes in Sustainability Competitive Advantage (SCA) *via* Behavioral Inertia (INT) moderated by Empowering Leadership (EL). Using a 5,000-bootstrap sample, we obtained a 95 percent bootstrap confidence interval with an indirect effect bias.

The *t*-count value of cynicism toward changes to inertia (INT) was −0.964 and the *p*-value was 0.224. Hypothesis 1 thus fails. The second hypothesis states that inertia reduces SCA. The t-count is 0.449 with a *p*-value of 0.657, indicating that hypothesis 2 is unsupported. The third hypothesis predicts that EL will reduce cynicism’s impact on family business inertia. The moderating variable (CynDisp*EL) has a t-value of 2.426 and a *p*-value of 0.17, supporting the hypothesis.

**Figure 1:**
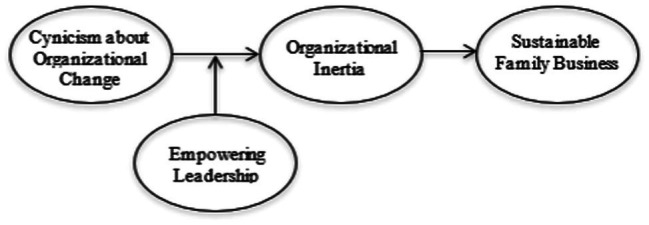
Conceptual model of the study.

Cynicism has an indirect effect on SCA changes *via* inertia, according to Hypothesis 4. For H3, Figure 1 and Table 3 show the moderated mediation of Hayes’ 7 model outputs. Model 1’s outcome variable has a 38.50 percent variation (Inertia). The model fits with a *F* value of 5.630. EL has a significant positive effect on inertia, with a *p*-value of 0.039 0.05. The LLCI and ULCI are not zero because of the significant interaction (int 1 = Cyndisp*EL). This suggests that EL does act as a moderator in the relationship between cynicism about organizational change and organizational inertia (Hayes, 2013).

**Table 3:** Model summary and coefficient (Hayes v3.5) Model 7.

Model summary	Model		*R*		*R* ^2^		MSE		*F*		*p*		Outcome
	Model 1		0.620		0.385		0.187		5.630		0.039		Inertia
	Model 2		0.621		0.386		0.152		8.807		0.001		SCA
Coefficient	Model		Coeff		*t*		*p*		LLCI		ULCI		Outcome
	Constant		2.410		26.948		0.000		2.226		2.594		INT
	CynDisp		−0.198		−0.964		0.344		−0.620		0.224		INT
	EL		−0.308		−2.465		0.020		−0.565		−0.052		INT
	DispCyn*EL		−0.884		−2.426		0.017		0.389		1.380		INT
	Constant		5.819		17.861		0.000		5.152		6.486		SCA
	Inertia		0,063		0.449		0.657		−0.225		0.352		SCA
	CynDisp		−0.585		−4.135		0.000		−0.875		−0.295		SCA
Effect		SE	*T*	*p*	LLCI	ULCI	Md	EL	Effect	B_SE	LLCI	ULCI	Index
Direct	0.063	0.141	0.448	0.657	−0.225	0.352							
In-direct							INT	−0.868	0.565	0.301	−0.020	1.19	
							INT	0.000	0.116	0.157	−0.211	0.428	
							INT	0.868	−0.324	0.143	−0.605	−0.041	
Moderation-mediation index										0.203	−0.937	−0.167	−0.517

The result of the mediation model is as follows: SCA is the criterion and Cyndisp is the independent variable. The proposed model’s R2 is 38.600%, *F* is 8.807, and *p*-value (0.001) is significant. Since Inertia directly affects SCA, it appears to be a mediator in the relationship between Cyndisp and SCA. Also, the LLCI and ULCI Boot values are both negative, with no zeros between them. So EL is a moderator at low, average, and high levels.

**Figure 2:**
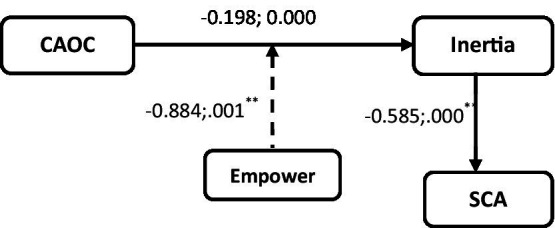
Hayes v3.5 Model-7 output statistical diagram. **Correlation values are significant at *p* < 0.01.

**Figure 3:**
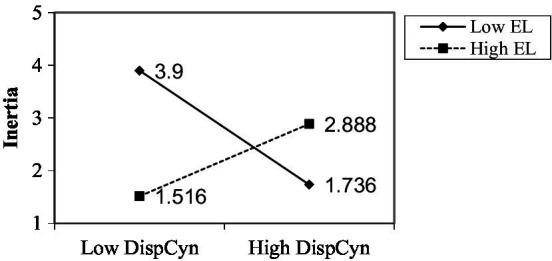
The effect of cynicism towards change on inertia at various levels of empowering leadership.

An interaction plot is made to see if the interaction is in the predicted direction. As shown in Figure 2, when leaders are empowered and cynical about change, the inertia value is moderate. Moderate inertia indicates that the family business can maintain a competitive advantage while maintaining the status quo. When cynicism toward change is high and the value of empowered leadership is low, inertia tends to be valuable. Due to the low inertia, the family business is more likely to be dynamic in the long run, thereby establishing a sustainable competitive advantage. Additionally, when cynicism toward change is low and empowerment of leadership is low, it has been demonstrated that the value of inertia is low. Inertia results from low cynicism toward change and high empowerment. Inertia indicates a family business is keeping things the same (Figure 3).

Because of the interaction between cynicism about organizational change and empowering leadership, the result demonstrates the ability of the interaction to produce suppression and buffering as a reciprocal triadic mechanism in Social Cognitive Theory (Bandura and Wood, 1989; Frazier et al., 2004; Li and Yuan, 2017; Lorinkova and Perry, 2017). Cynicism about organizational change, as indicated by a reduction in the regression coefficient value of cynicism about organizational change, from −0.884 to −0.198 in the interaction regression coefficient, is said to be able to suppress by the interaction when it can suppress by the interaction (Frazier et al., 2004; Li and Yuan, 2017). Organizational inertia is reduced by the positive influence of empowering leadership, which acts as a buffer against the negative impact of cynicism about organizational change. In other words, when empowering leadership by the leader is perceived as creating a positive environment for employees, the relationship between cynicism about organizational change and organizational inertia is reduced (Lorinkova and Perry, 2017).

## Discussion and Implication

The findings have many theoretical implications: To begin, research shows that leaders who empower employees are less effective. The results show that EL is only effective at high concentrations to reduce DispCyn’s negative effect on inertia. Perhaps the most important theoretical contribution of this study is expand the use of Social Cognitive Theory in the context of family business, besides the results of this study indicate that empowering leadership is a form of environmental in triadic reciprocal (Bandura, 1989; Amundsen and Martinsen, 2014; Lim et al., 2020). It has been proven that a positive environment of change can be a suppressor in suppressing cynicism for change, besides that a positive environment can also be a buffer in suppressing the negative influence of cynicism on changes to organizational inertia (Buffer). EL and cynicism change only when subordinates have positive empowering exchange relationships with superiors. Thus, the moderated-mediated model assumes a fully moderated negative relationship between EL and cynicism. These findings add to the growing body of research on the impact of leadership empowerment by highlighting the critical role of empowerment in generating exchange (Lorinkova and Perry, 2017).

The following are the implications: The first use of empowering leadership is to increase employee psychological empowerment and reduce cynicism about change. However, it is important for family business owners to remember that employees must psychologically feel empowered by the owner or leader. The effects of dyadic relationships can be felt by frontline employees, so direct supervisors and their supervisors are encouraged to cultivate high-quality dyadic relationships. This study suggests that family businesses actively train members to manage with the EL style through training and coaching. Development assistance can help reduce cynicism and inertia (Kim et al., 2018a,b).

Second, in the context of family business changes that place employees under pressure, discomfort, and/or uncertainty (Dhaenens et al., 2018; Lorenzo Gomez, 2020), leaders must position themselves as role models for employees, particularly cynical employees. Employees will learn from their leaders how to adapt to change, modify their behavior, and combat cynicism. By reducing employee cynicism through empowering leadership behaviors demonstrated by a leader who also enjoys a positive relationship with top management, managers can ensure a happier workplace and possibly even a more seamless transition to a new organizational reality without experiencing inertia (Santiago, 2015; Hirschmann, 2021).

## Limitation and Future Research

This study has various limitations, including the following: (1) the use of cross-sectional data, (2) the lack of a research gap between variables, (3) data processing at the same place, and (4) the inability to conduct simultaneous testing due to the dimensions of the test equipment utilized. Because of this, it is recommended that longitudinal data be used in the next study. This is done in order to ensure that there is a gap between CAOC and SCA in terms of influencing inertia. Researchers can use a time lag of 3–6 months with the same respondents in order to get more accurate results. Furthermore, researchers can use covariance-based SEM to determine whether or not a test is unidimensional.

## Conclusion

The findings of this study indicate that cynicism toward organizational change has a beneficial and statistically significant effect on organizational inertia. Additionally, empowering leadership has a negative moderating effect on the relationship between cynicism about organizational change and organizational inertia. Overall, this study sheds new light on the importance of empowering leadership in family businesses in suppressing members’ cynicism toward change, thereby reducing the likelihood of organizational inertia. A leader’s action in creating a favorable environment for initiated changes can also be defined as “a leader’s action in facilitating the implementation of changes” (Lorinkova and Perry, 2017). When empowered leadership suppresses cynicism about change, it is doing so in the form of suppression (Frazier et al., 2004; Li and Yuan, 2017). It is anticipated that the interaction of the two variables will act as a buffer, mitigating the negative impact of cynicism on changes in organizational inertia by at least a factor of two (Huang et al., 2013). The results of the moderated mediation test revealed that EL was responsible for determining the indirect effect of CAOC on SCA through organizational inertia in the study. EL not only reduces CAOC (Suppress), but it also supports the relationship between CAOC and inertia (Buffer), and it determines the indirect effect of CAOC on SCA through inertia (Frazier et al., 2004; Amundsen and Martinsen, 2014; Hayes, 2018).

## Data Availability Statement

The raw data supporting the conclusions of this article will be made available by the authors, without undue reservation.

## Ethics Statement

Ethical review and approval was not required for the study on human participants in accordance with the local legislation and institutional requirements. The patients/participants provided their written informed consent to participate in this study.

## Author Contributions

TT and VS presented the idea about how cynicism might affect the sustainability of an organization. SS and EA focused on how to conduct the data analysis with the multi-source and data aggregation, while TS and EA helped to gain access to family business networks in Indonesia. TT supervised the progress of this paper and added some references about organizational change and cynicism. VS helped to add some contribution and discussion to this paper. Finally, TT and EA edited the manuscript. All authors contributed to the article and approved the submitted version.

## Conflict of Interest

The authors declare that the research was conducted in the absence of any commercial or financial relationships that could be construed as a potential conflict of interest.

## Publisher’s Note

All claims expressed in this article are solely those of the authors and do not necessarily represent those of their affiliated organizations, or those of the publisher, the editors and the reviewers. Any product that may be evaluated in this article, or claim that may be made by its manufacturer, is not guaranteed or endorsed by the publisher.
